# Editorial: From biomass to bio-energy and bio-chemicals: Pretreatment, thermochemical conversion, biochemical conversion and its bio-based applications

**DOI:** 10.3389/fbioe.2022.975171

**Published:** 2022-10-28

**Authors:** Chao Zhao, Zhongqing Ma, Caoxing Huang, Jialong Wen, Muhammad Hassan

**Affiliations:** ^1^ National Engineering Research Center for Wood-based Resource Utilization, College of Optical, Mechanical and Electrical Engineering, Zhejiang A&F University, Hangzhou, China; ^2^ College of Chemistry and Materials Engineering, Zhejiang A&F University, Hangzhou, China; ^3^ Co-Innovation Center for Efficient Processing and Utilization of Forest Resources, College of Chemical Engineering, Nanjing Forestry University, Nanjing, China; ^4^ Beijing Key Laboratory of Lignocellulosic Chemistry, Beijing Forestry University, Beijing, China; ^5^ U.S.-Pakistan Centre for Advanced Studies in Energy, National University of Science and Technology (NUST), Islamabad, Pakistan

**Keywords:** pretreatment, lignocellulose fractionation, enzymatic hydrolysis, genetic engineering, bio-chemicals

Lignocellulosic biomass is the most abundant renewable feedstock on the Earth, which can be used to produce bio-fuels and bio-chemicals to support the sustainable development (Chundawat et al., 2011). Owing to the dual challenges of limited resources and the impacts on the environment, research on the efficient preparation of energy and chemicals using lignocellulosic biomass has attracted considerable attention (Lin et al., 2021).

Bio-energy derived from biomass is one of the most important renewable energy sources. It includes three major types occurring in three states: solid (briquette), liquid (biodiesel, bioethanol, and bio-oil), and gaseous (biogas, syngas, and biohydrogen) (Yu et al., 2021). There are broadly two conversion platforms for biomass: thermochemical conversion and biochemical conversion (Zhao et al., 2020a). Pretreatment is a necessary procedure for obtaining homogeneous raw materials. However, there remain many challenges including biomass heterogeneity resulting from the variation in biomass components, as well as the structural variations in each constituent, complicated pretreatment processes, low conversion efficiency, the high cost of enzymatic hydrolysis, the efficient engineering of bacteria breeding, and the application of its by-products (biochar for thermochemical conversion and lignin for biochemical conversion) (Qiao et al., 2018).

Bio-chemicals with excellent performance and high value added can be produced from biomass through physical, chemical, and biological high-tech methods. Similarly, the structure and properties of biomass limit the separation, purification, and effective utilisation of its components. There are still many challenges in the preparation of bio-chemicals from biomass, notably the selection of chemical reaction paths derived from multiscale complexity, the highly-selective depolymerisation, deoxygenation, polymerisation inhibition, and repolymerisation reactions of biomass components, especially the selective fracture of chemical bonds between cellulose, hemicellulose, and lignin (Zhao et al., 2020b).

The topic *From biomass to bio-energy and bio-chemicals: Pretreatment, thermochemical conversion, biochemical conversion and its bio-based applications* covers sustainable bio-refining processes from biomass to chemicals, sustainable pretreatment/fractionation technologies from biomass to bio-energy, genetic engineering of lignocellulosic feedstocks for bio-refinery, and sustainable bio-based applications in architecture, electrode materials, and adsorbents. We sincerely thank the 109 authors whose work we have drawn on for their excellent works on this topic. The following highlights have been drawn from their contributions to this Research Topic.

## Sustainable bio-refining processes from biomass to chemicals

Zhou et al. evaluated the effects of ultrasound-assisted production of xylo-oligosaccharides (XOS) from alkali-solubilised corncob bran. The maximum yield of XOS was 20.71% at the ultrasonic parameters of 50 kHz and 0.40 W/cm^2^. Lv et al. proposed a new reaction pathway for the synthesis of functional hydroxy-methyl-furfural (HMF) derivatives (EMFM, BHMFM, and BHMFD) from commercially available furfural derivatives (EMF and FA), and provided a new method to transform condensed furanics into hydrocarbon fuel through a two-step hydro-deoxygenation (HDO) process. Although such a pathway is feasible, achieving high yields of hydroxymethylated products in these reactions is challenging, owing to the parallel ring-opening reaction of furanics and condensation of the resulting ring-opening products. Therefore, future research should focus on the development of more efficient catalysts or reaction systems to suppress these side reactions and improve the selectivity of the targeted products. Korkalo et al. developed a cascade processing scheme to prepare pyroligneous acids (PAs) from the extractives of hybrid aspen bark. To obtain PAs, firstly the lipophilic and hydrophilic extracts were extracted through pretreatment of the bark using hot water and alkaline alcohol (HWE+AAE), and then the extractives were torrefied to PAs. The PAs obtained showed excellent herbicide and fungicide performances.

As the only renewable carbon source, biomass can be converted into chemicals with great potential prospect. In recent years, many advances have been made in the preparation of chemicals from biomass; however, the majority of these are still in the laboratory research stage and cannot meet the requirements of large-scale production. Future research should focus on the following aspects: 1) strengthening the research on the reaction mechanisms and pathways of biochemical conversion; 2) further optimisation of the catalytic system and the reaction conditions to improve the yield and selectivity of target products; and 3) establishing an efficient, low-energy, low-cost and environmentally friendly product separation system. With the deepening of basic research, the large-scale production of highly value-added chemicals from biomass is becoming promising.

## Sustainable pretreatment/fractionation technologies from biomass to bio-energy

Nawaz et al. explored the sustainable pretreatment of sawdust with levulinic acid-based natural deep eutectic solvents (DESs). The optimised molar ratio of levulinic acid: choline chloride was 1: 0.5, which resulted in 91% delignification and 25.87% maximum enzymatic hydrolysis of the sawdust. Fermentation produced 11.82% of bioethanol production was obtained at 30°C, and 180 rpm after 72 h. Lv et al. explored the use of poly (N-vinylcaprolactam) (PNVCL) to improve the enzymatic hydrolysis of bamboo after pretreatment with phenyl-sulfonic acid (PSA) pretreatment. A cellulosic conversion of 80% of PSA-treated-bamboo was achieved when the PNVCL loading was 1.2 g/L during enzymatic hydrolysis. Compared to the case without the addition of PNVCL, the cellulase loading of PSA-treated-bamboo was three times reduced. Mechanism research has shown that PNVCL can effectively prevent non-productive adsorption of enzymes through inter-molecular non-covalent interactions. Liu et al. isolated lignin from tobacco stalks using a hydrothermally assisted dilute alkali pretreatment. Subsequently, the isolated alkaline lignin was fractionated into five uniform lignin components using various solvents. According to the different structures of lignin fractions, Liu et al. suggested that lignin fractions with lower molecular weights were more suitable for preparing antioxidants, whereas those with high molecular weights showed great potentials for preparing carbon materials.

For biochemical conversion route, the goal of the pretreatment is to improve the enzymatic hydrolysis of cellulose. Sustainable pretreatment technologies include green solvent-based ionic liquid and DESs. In addition, future pretreatment research should focus on the modification of traditional pretreatments, addressing aspects such as biocatalytic methods, enzyme properties and enzyme recycling, making full use of lignin valorisation, and the management of residual streams from the perspective of biorefinery.

## Genetic engineering of lignocellulosic feedstocks for bio-refinery

Peng et al.studied the effects of *p*-coumarate 3-hydroxylase (C3H) down regulation on the chemical and structural characteristics of hemicelluloses and lignin, and found that the down-regulated poplar wood is beneficial for the upstream gene validation and downstream biomass conversion. Tienaho et al. studied the functional profiles of 16 northern willow clones for the use of value-added bioactive solutions**.** The results showed that all of the clones had antibacterial activity against *Staphylococcus aureus* and *Escherichia coli*, but no antifungal (*Aspergillus brasiliensis*) or yeasticidal (*Candida albicans*) efficacy. Additionally, *S. myrsinifolia* clone extracts showed significantly higher activities in some antioxidant tests than the commercial clone extracts and artificial clone extracts.

Both genetic modification and pretreatment are expected to reduce biomass recalcitrance, and genetic modification of plants is considered to be ultimate solution to reduce biomass recalcitrance. At present, the majority of modifications use lignin gene manipulation to reduce biomass recalcitrance, such as down-regulation of the switchgrass caffeic acid O-methyltransferase gene, or down-regulation of cinnamyl alcohol dehydrogenase which leads to incorporation of aldehyde groups in the lignin polymer. The potential for the use of atomistic molecular dynamics (MD) simulations as a predictive tool for the effect of genetic modifications on plants should be pursued in future work. This knowledge paves the way towards the development of high value-added biochemicals and other functional solutions based on genetic engineering of lignocellulosic feedstocks for biorefinery approaches.

## Sustainable bio-based applications from biomass

Wang and Zhu discussed the research progress of biomass-based transparent wood (BBTW), and summarized the key technologies and potential prospects of BBTW for the replacement of architectural glass. As a green and renewable material, BBTW offers the advantages of good lighting conditions, flame retardancy, heat insulation and safety. Hu et al. designed the lignin/polypyrrole (PPy) composite electrode films with microporous and mesoporous structures by electrostatic spinning, carbonisation, and *in-situ* polymerisation methods, which can be served as a positive material for super-capacitors. The LCNF/PPy electrode had a large specific surface area, high pore volume, and a specific capacitance of 213.7 F/g at a current density of 1 A/g. Shi et al. studied the adsorbents produced using multifunctional cellulose and cellulose-based (nano) composites. The authors believed that these biomass adsorbents will play an increasingly important role in environmental protection, particularly in wastewater treatment.

Nano-cellulose has many advantages such as high modulus, high specific surface area, special optical properties and good bio-compatibility. Nano-lignin has the characteristics of a highly specific surface area, multiple chemical groups, antibacterial activity, and non-toxicity. As a green and environmental bio-based material, nano-lignocellulose has broad applications in architecture, food industry, biomedicine, environmental materials, photoelectric materials, and other fields. However, more research is needed to explore the efficient preparation of homogeneous and stable nanolignin. For nanocellulose, promoting the development of products with both cost and performance characteristics and developing green and environmentally friendly preparation methods could accelerate their industrialisation and large-scale production processes.
